# HtrA-mediated E-cadherin cleavage is limited to DegP and DegQ homologs expressed by gram-negative pathogens

**DOI:** 10.1186/s12964-016-0153-y

**Published:** 2016-12-08

**Authors:** Carmen M. Abfalter, Maria Schubert, Camilla Götz, Thomas P. Schmidt, Gernot Posselt, Silja Wessler

**Affiliations:** Division of Microbiology, Department of Molecular Biology, Paris-Lodron University of Salzburg, Billroth Str. 11, A-5020 Salzburg, Austria

**Keywords:** HtrA, DegP, DegQ, E-cadherin

## Abstract

**Background:**

The serine proteases HtrA/DegP secreted by the human gastrointestinal pathogens *Helicobacter pylori* (*H. pylori*) and *Campylobacter jejuni* (*C. jejuni*) cleave the mammalian cell adhesion protein E-cadherin to open intercellular adhesions. A wide range of bacteria also expresses the HtrA/DegP homologs DegQ and/or DegS, which significantly differ in structure and function.

**Methods:**

E-cadherin shedding was investigated in infection experiments with the Gram-negative pathogens *H. pylori*, enteropathogenic *Escherichia coli* (EPEC), *Salmonella enterica* subsp. Enterica (*S.* Typhimurium), *Yersinia enterocolitica* (*Y. enterocolitica*), and *Proteus mirabilis* (*P. mirabilis*), which express different combinations of HtrAs. Annotated wild-type *htrA*/*degP*, *degQ* and *degS* genes were cloned and proteolytically inactive mutants were generated by a serine—to—alanine exchange in the active center. All HtrA variants were overexpressed and purified to compare their proteolytic activities in casein zymography and in vitro E-cadherin cleavage experiments.

**Results:**

Infection of epithelial cells resulted in a strong E-cadherin ectodomain shedding as reflected by the loss of full length E-cadherin in whole cell lysates and formation of the soluble 90 kDa extracellular domain of E-cadherin (NTF) in the supernatants of infected cells. Importantly, comparing the caseinolytic and E-cadherin cleavage activities of HtrA/DegP, DegQ and DegS proteins revealed that DegP and DegQ homologs from *H. pylori*, *S.* Typhimurium, *Y. enterocolitica*, EPEC and *P. mirabilis*, but not activated DegS, cleaved E-cadherin as a substrate in vitro.

**Conclusions:**

These data indicate that E-cadherin cleavage is confined to HtrA/DegP and DegQ proteins representing an important prevalent step in bacterial pathogenesis.

**Electronic supplementary material:**

The online version of this article (doi:10.1186/s12964-016-0153-y) contains supplementary material, which is available to authorized users.

## Background

Human pathogens developed sophisticated strategies to survive and colonize under extreme conditions or to conquer host defense mechanisms. The serine proteases HtrA/DegP are important key players in protein quality control and stress response through refolding and degrading misfolded proteins in the periplasm of bacteria [1, 2]. In *E. coli*, DegP was identified as an ATP-independent heat shock protease that maintains protein homeostasis in the periplasm by combining chaperone and protease activities. DegP consists of an N-terminal signal peptide, which is responsible for its periplasmic localization followed by a conserved chymotrypsin—like protease domain harboring the catalytic triad composed of a histidine, a serine and an aspartate residue. In the C-terminal region, DegP contains two flexible PDZ (postsynaptic density protein [PSD95], *Drosophila* disc large tumor suppressor [Dlg1], and zonula occludens-1 protein [ZO-1]) domains mediating protein-protein interactions, substrate recognition and substrate binding [1, 3, 4]. The monomeric *E. coli* DegP can form trimers, hexamers, dodecamers, and finally active 24-mers [5, 6]. It was demonstrated that binding of hexameric DegP to misfolded proteins leads to the formation of active 12-mers and 24-mers [5]. Several substrates for *E. coli* DegP were described, including maltose binding protein, alkaline phosphatase, α-amylase, outer membrane protein OmpF and OmpC, the pilin subunit PapA or the acylated precursor of colicin A lysis protein [2]. *E. coli* also expresses the HtrA/DegP homologs DegQ (HhoA, HtrA homolog A) and DegS (HhoB, HtrA homolog B). The main difference between DegP and DegQ is the length of the N-terminally positioned LA loop, which lacks 20 amino acids in DegQ [7, 8]. The LA loop is implicated in the stabilization of the inactive hexameric conformation of DegP [9]. Although DegQ and DegP exhibit ~60% sequence identity, it is not fully understood whether they share overlapping function. It was shown that DegQ is capable of rescuing temperature sensitive *degP*-negative strains [10], while others have postulated that the substrate specificity of DegQ might be different since DegQ re-expression could not fully restore the phenotype of a *degP* knock-out mutant [8, 11]. DegS is considered as a regulatory protease targeting the anti-sigma factor RseA in the periplasm, which is implicated in sensing protein folding stress. After detecting misfolded outer membrane proteins, DegS processes the anti-sigma factor RseA, which is followed by RseP cleavage. As a regulated intramembrane proteolysis cascade, this leads to the sigma-E-mediated expression of factors involved in protein folding stress in the periplasm and assembly of outer membrane proteins [3, 12].

In many pathogenic bacteria, HtrA promotes virulence as reflected by the observation that *htrA* knock-out mutants show either an apathogenic phenotype or a significantly reduced virulence [2, 13]. A widespread explanation for the HtrA-dependent pathogenesis arose from the observation that HtrA increases bacterial survival under stress conditions during infection. Further, it was suggested that HtrA is involved in the processing of outer membrane (virulence) factors [13]. For instance, DegP was identified as a critical factor for IcsA (VirG) surface presentation in *Shigella flexneri* (*S. flexneri*) [14]. Furthermore, reduced adherence of a *C. jejuni htrA* knock-out mutant was observed in vitro [15–18] suggesting that the expression of adhesins might be downregulated. However, in a mouse model for *C. jejuni* infections, isogenic *htrA*-negative bacteria colonized equally well, while host cell apoptosis and the pro-inflammatory immune responses were significantly attenuated [19, 20]. Similar observations were made for a number of other *htrA*-negative pathogens in vivo (e.g. *Yersinia pestis*, *Streptococcus pneumoniae*, *Mycobacterium tuberculosis, Listeria monocytogenes, Klebsiella pneumoniae, etc.*) [13, 21]. In *Chlamydia trachomatis* (*C. trachomatis*) HtrA functions as an active chaperone and serine protease [22]. HtrA is secreted from chlamydial inclusions into the host cytoplasm independently of the type-III secretion system [23] and exhibits a critical role in the replicative phase of the chlamydial developmental cycle [24]. These data underline the crucially important role of HtrA in bacterial pathogenesis. However, the molecular mechanism remained largely unknown.

An additional function of HtrA in several Gram-negative pathogens of the gastrointestinal tract was recently described. During infection with *H. pylori* and *C. jejuni*, HtrA is secreted into the microenvironment [25, 26] and was detected in outer membrane vesicles (OMVs) [27, 28]. *H. pylori* and *C. jejuni* HtrAs cleave-off the extracellular domain of the cell adhesion protein E-cadherin on epithelial cells [15, 27, 29, 30]. E-cadherin is an important key molecule in the establishment and maintenance of an intact epithelial barrier. Consequently, E-cadherin cleavage disrupts the barrier function and allows bacterial entry into the intercellular space and transmigration [31, 32]. In *H. pylori* or *C. trachomatis*, genomic htrA deletions mutants could not be generated so far. However, functional small molecule inhibitors and substrate-derived peptide inhibitors were designed which efficiently blocked HtrA functions [30, 33, 34]. HtrA-mediated E-cadherin cleavage was also shown for EPEC and *S. flexneri* supporting our hypothesis that E-cadherin ectodomain shedding might be a prevalent mechanism for pathogenic bacteria to promote virulence through the interference with (baso-) lateral domains of epithelial cells [15]. However, these studies were restricted to HtrA/DegP and the role of DegQ and DegS in E-cadherin cleavage was not considered so far. In this report, we investigated E-cadherin shedding in response to infection with the Gram-negative gastrointestinal pathogens *H. pylori*, EPEC, *Y. enterocolitica*, *S. enterica* subsp. Enterica (*S.* Typhimurium) and the uropathogenic bacterium *P. mirabilis*, which express different combinations of HtrA proteins.

## Methods

### Infection experiments

MKN-28 and NCI-N87 cells were grown in RPMI 1640 medium (Sigma Aldrich) containing 10% FBS (Sigma Aldrich) in 6-well plates to a confluency of 70 to 80% for 2 days. 16 h prior to the infection, medium was replaced by serum-free RPMI 1640. *H. pylori* (Hp26695) was cultivated on GC-Agar plates containing 10% horse serum under microaerophilic conditions (CampiGen, Thermo Scientific) at 37 °C for 2 days. *P. mirabilis* (ATCC 29906) was grown on nutrient agar, and EPEC (E2348), *Salmonella enterica* subsp. Enterica (*S.* Typhiumurium, NCTC 12023) and *Y. enterocolitica* (ATCC 27729) were cultivated on LB agar plates for 24 h at 37 °C. Serum-starved cells were infected at a multiplicity of infection (MOI) of 100 with *H. pylori*, at a MOI 5 with EPEC or *S.* Typhimurium*,* at a MOI 50 with *Y. enterocolitica* and at a MOI 2 with *P. mirabilis*. Cells were harvested after indicated time periods in lysis buffer (20 mM Tris pH 7.5, 1 mM EDTA, 100 mM NaCl, 1% Triton X-100, 0.5% DOC, 0.1% SDS, 0.5% NP-40). Samples were centrifuged for 10 min at 16000 × g at 4 °C. Pellets were discarded and lysates were analyzed for full length E-cadherin by Western blotting. For the detection of the soluble extracellular E-cadherin fragment, supernatants of infected cells were collected. Bacteria were harvested in sterile PBS supplemented and sonicated to prepare bacterial lysates. Protein amounts were measured using Bradford (RotiQuant, Carl Roth).

### SDS PAGE and western blotting

10 μg of the bacterial lysates or 0.5 μg recombinant proteins were separated by SDS-PAGE and stained using 1% Coomassie Brilliant Blue G250 (BioRad). To investigate E-cadherin cleavage, 50 μg of cell lysates or 100 μl of supernatants were separated by SDS-PAGE and transferred onto nitrocellulose membranes. Monoclonal antibodies recognizing the extracellular domain (ab40772, Abcam) or intracellular domain (24E10, Cell Signaling) of E-cadherin were used to detect the NTF in supernatants or the loss of full length E-cadherin in whole cell lysates, respectively. ß-actin was detected using a monoclonal antibody (Sigma Aldrich).

### Casein zymography

10 μg of the bacterial lysates or 1 μg recombinant proteins were separated by casein-containing SDS gels under non-reducing conditions. Subsequently, gels were renatured in 2.5% Triton X-100 and equilibrated in developing buffer as previously described [25]. Caseinolytic activity was visualized after staining with 0.5% Coomassie Blue R250 (BioRad).

### Sequence analysis

Protein sequences from *H. pylori* HtrA (G2J5T2), EPEC DegP (B7UIK8), EPEC DegQ (B7UJW6), EPEC DegS (B7UJW7), *S.* Typhimurium HtrA (P26982), *Y. enterocolitica* DegP (P74978), *P. mirabilis* DegQ (B4EXL6), *P. mirabilis* DegS (B4EXL5) were retrieved from UniProt (Table 1). Sequence alignments were performed using Clustal Omega [35]. Protein domain prediction was performed using SignalP4.1 and SMART (simple modular architecture research tool) [36–38]. (*) indicates identical amino acids in all sequences, conserved amino acid substitutions are labeled with (:) and semi-conservative substitutions are marked with (.).

**Table 1:** Proteins analyzed in this study

Pathogen	Strain	Name	Uniprot^a^	Primer sequences^b^	Mutagenesis primer^c^	Reference
*Helicobacter pylori*	Hp26695	HtrA	G2J5T2	5′-AAGGATCCGGCAATATCCAAATCCAGAGCATG-3′ 5′-AAGAATTCGACCCACCCCTATCATTTCACC-3′	5′-GCTTCCATCAATCCTGGAAATGCTGGCGGCGCTTTAATTGATAGC-3′ 5′-GCTATCAATTAAAGCGCCGCCAGCATTTCCAGGATTGATGGAAGC-3′	[25]
EPEC	E2348/69	DegP	B7UIK8	5′-GGATCCGCTGAGACTTCTTCA-3′ 5′-CCCGGGTTACTGCATTAACAG-3′	5′-CAACCGGGGTAACGCAGGTGGTGCGTTG-3′ 5′-CAACGCACCACCTGCGTTACCCCGGTTG-3′	[15]
		DegQ	B7UJW6	5′-GATCGGATCCATTCCAGGCCAGGTTGCCGC-3′ 5′-CTAGCTCGAGTAACGCATTAGTAGGTAGAG-3′	5′-CATTAACCGCGGTAACGCCGGCGGTGCACTGTTAAAC-3′ 5′-GTTTAACAGTGCACCGCCGGCGTTACCGCGGTTAATG-3′	This work
		DegS	B7UJW7	5′-GATCGGATCCAGCCTTAACCCGCTTTCCAC-3′ 5′-CTAGGAATTCTTAGTTGGTCGCCGGATATT-3′	5′-CCATTAACCACGGTAACGCTGGCGGCGCGCTGG-3′ 5′-CCAGCGCGCCGCCAGCGTTACCGTGGTTAATGG-3′	This work
*Salmonella* Typhimurium	NCTC 12023	HtrA	P26982	5′-GATCGGATCCGCTGAAACGTCCTCTTC-3′ 5′-CATGCTCGAGTTACTGCATCAGCAAATAAATAG-3′	5′-CCGTGGTAACGCCGGCGGCGCGCTGG-3′ 5′-CCAGCGCGCCGCCGGCGTTACCACGG-3′	This work
*Yersinia enterocolitica*	ATCC 27729	DegP	P74978	5′-GATCGGATCCCCGGTTTCTTCTGTCGTTGC-3′ 5′-CTAGGAATTCTTACTGCATCAGCAGATAGAG-3′	5′-GCAATTAACCGTGGTAACGCCGGTGGTGCATTGATCAATC-3′ 5′-GATTGATCAATGCACCACCGGCGTTACCACGGTTAATTGC-3′	This work
*Proteus mirabilis*	ATCC 29906	DegQ	B4EXL6	5′-GGATCCGCCCTGCCTTCGGTAA-3′ 5′-GAATTCTTAACGCGAGCTGTTACGTAA-3′	5′-GCATCAATTAACCGTGGTAACGCTGGTGGTGCTTTAGTTAATC-3′ 5′-GATTAACTAAAGCACCACCAGCGTTACCACGGTTAATTGATGC-3′	This work
		DegS	B4EXL5	5′-GATCGAATTCATGTTAAGCAAGCTACTGCG-3′ 5′-CTAGCTCGAGCTATGACTCTGGCTGATATT-3′	5′-CAATTAATGAAGGAAATGCAGGGGGGGCACTGATTAATACTG-3′ 5′-CAGTATTAATCAGTGCCCCCCCTGCATTTCCTTCATTAATTG-3′	This work

*NCTC* National Collection of Type Cultures, *ATCC* American Type Culture Collection

^a^sequences of HtrA proteins; ^b^restriction recognition sites are underlined; ^c^substituted nucleotides are underlined

### Cloning, mutagenesis and protein purification

Cloning, mutagenesis and protein purification was performed as described before [25]. Briefly, genes encoding *H. pylori* HtrA (*Hp*HtrA aa 18–475), EPEC DegP (*Ep*DegP aa 27–474), EPEC DegQ (*Ep*DegQ aa 29–455), EPEC DegS (*Ep*DegS aa 28–355), *S.* Typhimurium HtrA (*St*HtrA aa 27–475), *Y. enterocolitica* DegP (*Ye*DegP aa 21–478), *P. mirabilis* DegQ (*Pm*DegQ aa 31–463), *P. mirabilis* DegS (*Pm*DegS aa 1–356) lacking predicted signal peptides were amplified. Primer sequences are shown in Table 1. PCR fragments flanked by restriction sites for BamHI/EcoRI (*Hp*HtrA, *Ep*DegS, *Ye*DegP and *Pm*DegQ), BamHI/XmaI (*Hp*HtrA) or *Eco*RI/*Xho*I (*Pm*DegS) were ligated into pGEX-6P-1 (GE Healthcare) for the expression of N-terminally tagged GST fusion proteins. Generation of inactive HtrA proteases (*Hp*HtrA^S221A^, *Ep*DegP^S236A^, *Ep*DegQ^S214A^, *Ep*DegS^S201A^, *St*HtrA^S237A^, *Ye*HtrA^S238A^, *Pm*DegQ^S219A^, *Pm*DegS^S199A^) was performed by S → A mutations in the active center using a site directed mutagenesis kit (Agilent) (Table 1). *E. coli* BL21 has been transformed with generated constructs and purification of the proteins was performed as previously described [25]. In brief, transformed *E. coli* was grown in 300 ml LB medium to an OD_600_ of 0.6 and the expression was induced by the addition of 0.1 mM isopropylthiogalactosid (IPTG). The bacterial culture was pelleted at 6000 × g for 30 min at 4 °C and lysed in 10 ml PBS by sonication. The lysate was cleared by centrifugation and the supernatants were incubated with glutathione sepharose (GE Healthcare Life Sciences) at 4 °C overnight. GST-tagged proteins were cleaved with 180 U Prescission protease (GE Healthcare Life Sciences) for 16 h at 4 °C to remove the GST tag. RseA (residues 121–216) fused to an N-terminal His_6_-tag was kindly provided by Tim Clausen (IMP, Vienna) and has been described previously [39]. RseA was expressed in *E. coli* BL21 and purified via ProBond NiNTA sepharose (Invitrogen). RseA was washed and eluted with 250 mM imidazole. All purified proteins were rebuffered in the respective cleavage buffer compatible with following cleavage experiments.

### Antibody production

A polyclonal antibody recognizing *Hp*HtrA was generated by the immunization of rabbits with recombinant *Hp*HtrA (Paul-Ehrlich Institute, Langen, Germany). Polyclonal antibodies for the detection of *St*HtrA, *Ye*DegP, *Ep*DegP or *Pm*DegQ were produced by immunization of rabbits with recombinant *St*HtrA^S237A^, *Ye*DegP^S238A^, *Ep*DegP^S236A^ and *Pm*DegQ proteins (David’s Biotechnology GmbH, Regensburg, Germany).

### In vitro cleavage assays

For in vitro cleavage studies, 50 ng of recombinant human E-cadherin (R&D) was incubated with 500 ng of recombinant proteases in 50 mM Hepes (pH 7.4) containing 1 mM EDTA at 37 °C for 24 h. As indicated, proteolytic inactive proteins were included as controls. Cleavage of E-cadherin was detected by Western blot analyses. To demonstrate *Ep*DegS activity, 7 μg *Ep*DegS was incubated with 9 μg recombinant RseA protein in the presence of 100 μM YFF (DNRLGLVYFF) activator peptide [40] for 16 h at 37 °C in 100 mM NaPO_4_ (pH 7.5), 200 mM NaCl, 5 mM MgCl_2_, 1 mM DTT und 10% glycerol. Where indicated, 300 ng E-cadherin was added. Aliquots of the samples were analyzed by Western blotting for E-cadherin cleavage, while RseA degradation was detected by coomassie-stained SDS PAGEs.

## Results

**Fig. 1:**
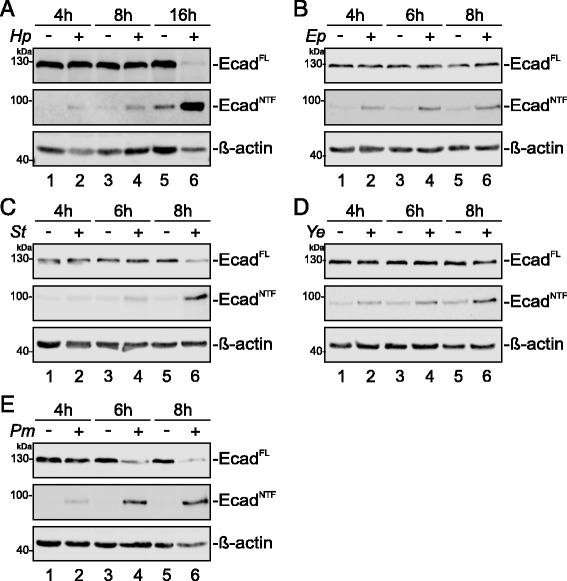
E-cadherin cleavage during infection with Gram-negative pathogens. Human epithelial cells were infected with (**a**) *H. pylori* (*Hp*) at a MOI 100, (**b**) EPEC (*Ep*) at a MOI 5, (**c**) *S.* Typhimurium (*St*) at a MOI 5, (**d**) *Y. enterocolitica* (*Ye*) at a MOI 50 and (**e**) *P. mirabilis* (*Pm*) at a MOI 2. Different MOIs were chosen after careful titration of infection doses to minimize bacterial overgrowth during infection. After indicated time periods, cells were lysed and full length E-cadherin (Ecad^FL^) was detected by Western blot analyses using an antibody against the intracellular domain. Aliquots of supernatants were analyzed for the soluble extracellular E-cadherin fragment (Ecad^NTF^) using an antibody against the extracellular domain. β-actin served as a loading control

*H. pylori* only harbors a DegP homolog, whereas EPEC, *S.* Typhimurium and *Y. enterocolitica* express DegP, DegS and DegQ, and the genome of *P. mirabilis* contains DegQ and DegS. To analyze their capacity to induce E-cadherin ectodomain shedding during infection, epithelial cells were colonized with selected pathogens and E-cadherin cleavage was investigated through detection of the loss of full length E-cadherin (E-cad^FL^) in whole cell lysates and the formation of the soluble N-terminal fragment (E-cad^NTF^) in the supernatants of infected cells. To demonstrate equal protein amounts in whole cell lysates, β-actin was shown. As reported previously [15], *H. pylori* (Fig. 1a) and EPEC (Fig. 1b) induced efficient E-cadherin shedding as monitored by increase of E-cad^NTF^ and, partially, by the corresponding decrease of E-cad^FL^ after indicated time periods of infection. Cells infected with *S.* Typhimurium showed an increased amount of the cleaved soluble E-cad^NTF^ in supernatants after 6 h and after 8 h. The amount of E-cad^FL^ detectable in cell lysates decreased correspondingly (Fig. 1c) indicating that infections with *S.* Typhimurium induces E-cadherin ectodomain shedding during infection as well. Similar observations were made for cells infected with *Y. enterocolitica* (Fig. 1d). Compared to non-infected cells, E-cad^FL^ slightly decreased, while E-cad^NTF^ in the supernatants of infected cells appeared (Fig. 1d). As a Gram-negative uropathogen, *P. mirabilis* was included in this study. *P. mirabilis* induced a very strong decline of E-cad^FL^ in whole cell lysates and correspondingly, the amount of E-cad^NTF^ drastically increased indicating an efficient cleavage of E-cadherin during colonization (Fig. 1e). These data imply that E-cadherin shedding occurs frequently during bacterial pathogenesis.

**Fig. 2:**
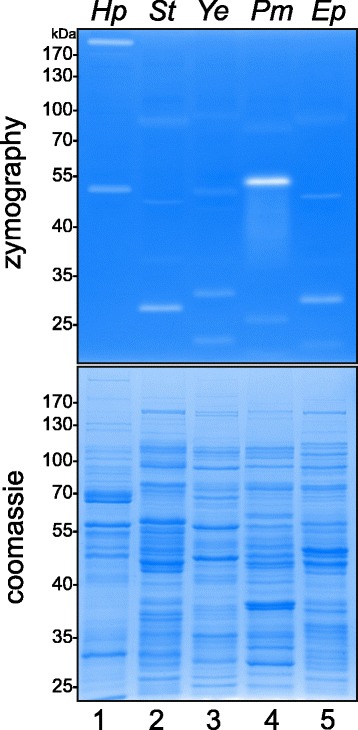
Active proteases expressed by pathogens. *H. pylori (Hp),* EPEC *(Ep)*, *S.* Typhimurium *(St)*, *Y. enterocolitica (Ye)* and *P. mirabilis (Pm)* were sonicated and protein lysates were analyzed by casein zymography (*upper panel*). As a control, proteins were separated by SDS PAGE followed by coomassie staining to show equal protein loading (*lower panel*)

E-cadherin shedding can be induced by host proteases [30, 41] or by bacterial proteases, such as HtrA proteins [15, 27, 30]. To evaluate if HtrAs of *S.* Typhimurium, *Y. enterocolitica*, or *P. mirabilis* are expressed and capable of E-cadherin cleavage, we analyzed the expression of proteolytic active proteases by casein zymography in a first step. *H. pylori* expressed caseinolytically active monomeric and oligomeric HtrA at 50 kDa and >170 kDa, which have been previously identified by mass-spectrometry [25]. In lysates of *S.* Typhimurium, three different activities at 85 kDa, 45 kDa and 28 kDa were observed. Four proteolytic activities (90 kDa, 55 kDa, 30 kDa and 20 kDa) were found in *Y. enterocolitica*, while *P. mirabilis* exhibited caseinolytically active proteases of approximately 75 kDa, 55 kDa and 25 kDa. EPEC lysates contained proteolytic activities at 50 kDa, 30 kDa and 20 kDa (Fig. 2, upper panel), of which the 50 kDa protease was identified as active DegP previously [15]. Equal protein amounts were demonstrated by a coomassie-stained SDS PAGE (Fig. 2, lower panel).

**Fig. 3:**
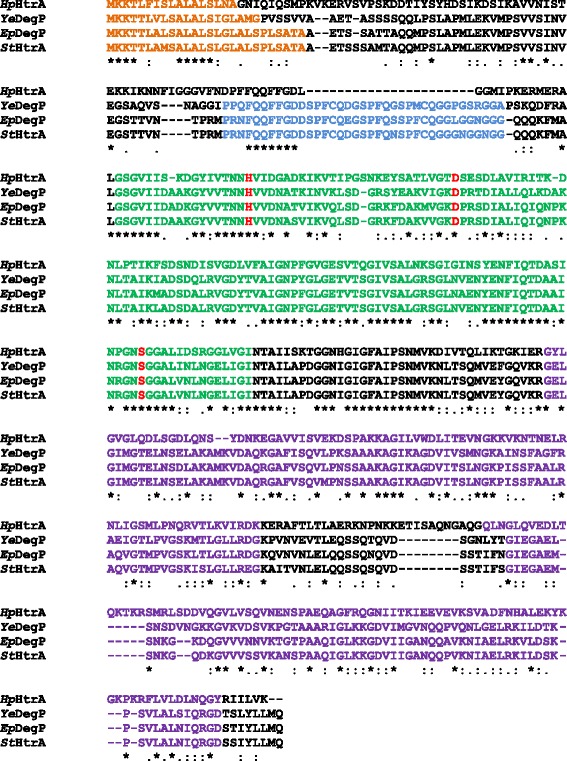
Sequence alignment of the different HtrA/DegP proteins. Signal peptides (*orange*), proteolytic domains (*green*) containing the catalytic triad (*red*) and two PDZ domains (*purple)* of *H. pylori* HtrA (*Hp*HtrA), *Y. enterocolitica* DegP (*Ye*DegP), EPEC DegP (*Ep*DegP) and *S.* Typhimurium HtrA (*St*HtrA) are indicated. The LA loop region is highlighted in *blue*

**Fig. 4:**
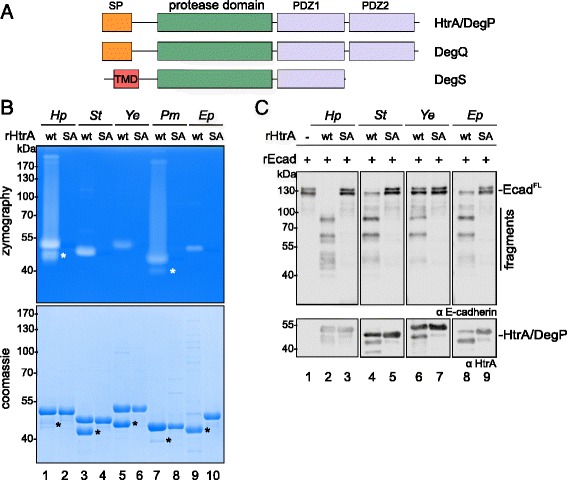
Recombinant HtrA’s/DegP’s are proteolytically active and cleave E-cadherin in vitro. a Domain architecture of HtrA/DegP, DegQ and DegS proteins. SP, signal peptide (*orange*); protease domain (*green*); PDZ domains (*purple*); TMD, transmembrane domain (*red*). **b** The proteolytic activity of recombinant HtrA/DegP (rHtrA) wildtype proteins (wt) of *H. pylori (Hp), S.* Typhimurium *(St)*, *Y. enterocolitica (Ye),* EPEC *(Ep)* and DegQ of *P. mirabilis (Pm)* was analyzed by casein zymography and compared to their corresponding inactive mutants (SA) (*upper panel*). Coomassie-stained SDS PAGEs demonstrated equal protein loading (lower panel). Self-processed proteins (*black asterisks*) exhibiting proteolytic activity (*white asterisks*) are indicated. **c** Recombinant HtrAs/DegPs (wt) were investigated in in vitro cleavage assays using E-cadherin (E-cad^FL^) as a substrate and compared with the corresponding inactive variants (SA) as a control. Fragments of E-cadherin were detected using an antibody recognizing the extracellular domain domain. HtrA/DegP proteins were detected using corresponding polyclonal antibodies

The serine protease HtrA or DegP is a highly conserved protease. Sequence alignments of HtrAs and DegPs of *H. pylori* (*Hp*HtrA), *Y. enterocolitica* (*Ye*DegP), EPEC (*Ep*DegP) and *S.* Typhimurium (*St*HtrA) demonstrated high similarities (Fig. 3). All HtrA/DegP proteases harbor a predicted N-terminal signal peptide (orange), a proteolytic domain (green) with the catalytic triad containing a histidine, an aspartate and a serine (red). The protease domain was followed by two C-terminal PDZ domains (purple). In comparison to DegQ proteases (Additional file 1: Figure S1A), the LA loop (blue) in DegP proteases contained additional 20 amino acids [7]. Interestingly, the LA loop of *H. pylori* HtrA lacked 22 amino acids suggesting that *H. pylori* HtrA might be a DegQ protein rather than a DegP protein. However, *Hp*HtrA shows a higher identity with *Ep*DegP (43% identity, E = 2e-87) compared to the alignment of *Hp*HtrA with *Ep*DegQ (37% identity, E = 8e-81), while a comparison of *Ep*DegQ and *Pm*DegQ uncovered an identity of 66% (Additional file 1: Figure S1A). DegS has a different domain architecture [1, 3]. DegS proteins often contain a transmembrane domain instead of a signal peptide and only one PDZ domain (Fig. 4a). Comparison of the amino acid sequence of *Ep*DegS and *Pm*DegS (Additional file 1: Figure S1B) showed an identity of 59% (E = 5e-144). However, a signal peptide has been predicted for *Ep*DegS and a putative transmembrane domain for *Pm*DegS (Additional file 1: Figure S1B), which might indicate that they also have different functions. To investigate the different bacterial HtrA/DegP proteins, we cloned, overexpressed and purified DegP proteins from *H. pylori* (*Hp*), *S.* Typhimurium (*St*), *Y. enterocolitica* (*Ye*), and EPEC (*Ep*) and analyzed the caseinolytic activity in casein zymography experiments (Fig. 4b). In *P. mirabilis* (*Pm*), DegP was not annotated; hence, the *degQ* gene was cloned. Additionally, proteolytic inactive proteases (*Hp*HtrA^S221A^, *Ep*DegP^S236A^, *St*HtrA^S237A^, *Ye*HtrA^S238A^, *Pm*DegQ^S219A^) were generated by the exchange of the serine by an alanine in the active center. Recombinant wildtype proteases (rHtrA^wt^) and their corresponding inactive mutants (rHtrA^SA^) were examined by casein zymography (Fig. 4b, upper panel) and coomassie-stained SDS PAGE (Fig. 4b, lower panel). In fact, all rHtrA^wt^ proteins were caseinolytically active to different extents. A strong activity was observed for *Hp*HtrA^wt^, *St*HtrA^wt^ and *Pm*DegQ^wt^, while *Ye*HtrA^wt^ and *Ep*DegP^wt^ were less active. As expected, the proteolytic inactive rHtrA^SA^ mutants did not show any activities (Fig. 4b, upper panel). In our previous studies, we already identified an auto-processed *H. pylori* HtrA (sHtrA, short HtrA) by mass-spectrometry [25] (Fig. 4b, lower panel, black asterisk), which was proteolytically active (Fig. 4b, upper panel, white asterisk). Auto-cleavage of DegP as part of a physiological process was also described for *E. coli* [42] and was also detected for *Ep*DegP in this study (Fig. 4b, lower panel). In contrast to *Hp*HtrA, auto-cleavage of *Ep*DegP was almost complete, but led to an inactivation of DegP. A similar picture was observed for *St*HtrA and *Ye*HtrA. Only the full length versions of *St*HtrA and *Ye*HtrA were proteolytically active, while the truncated proteins exhibited no activities. This is in a slight contrast to *Pm*DegQ. Comparable to *Hp*HtrA, we detected large amounts of active full length and a smaller fraction of active auto-processed *Pm*DegQ (Fig. 4b). These data imply that auto-proteolytic processing leads to an inactivation of *St*HtrA, *Ye*HtrA and *Ep*DegP, but not of *Hp*HtrA or *Pm*DegQ. Recombinant HtrA/DegP proteins were further used for the production of polyclonal antisera recognizing the individual proteins (Additional file 1: Figure S2). In order to evaluate their E-cadherin cleavage capability, purified DegP homologs (rHtrA^wt^) and the corresponding inactive mutants (rHtrA^SA^) were then examined in in vitro cleavage experiments using recombinant E-cadherin (rEcad) as a substrate. Incubation of rEcad with rHtrA/DegP from *H. pylori*, S. Typhimurium, *Y. enterocolitica* and EPEC induced the typical fragmentation pattern of rEcad indicating that the DegP homologs of the tested Gram-negative pathogens can directly target E-cadherin as a substrate. As expected, the inactive HtrA/DegP^SA^ proteins did not cleave rEcad (Fig. 4c). Polyclonal antibodies detecting the individual HtrA/DegP proteins (Additional file 1: Figure S2) showed equal loading of HtrA/DegP^wt^ and HtrA/DegP^SA^ proteins (Fig. 4c).

**Fig. 5:**
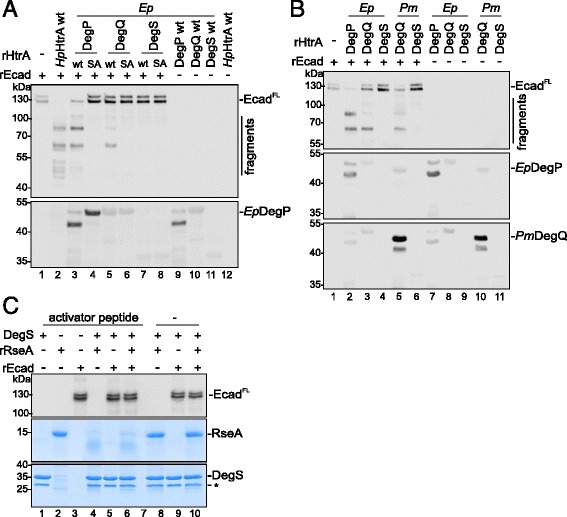
DegP and DegQ, but not DegS cleave E-cadherin in vitro. **a** DegP, DegQ and DegS wildtype (wt) of EPEC (*Ep*) and the corresponding inactive mutants (SA) were tested in in vitro cleavage assays using E-cadherin (rEcad) as a substrate (*upper panel*). *Ep*DegP^wt^ and *Ep*DegP^SA^ were detected using anti-*Ep*DegP antibody (*lower panel*). **b** The E-cadherin-cleavage activity of EPEC (*Ep*) DegP, DegQ and DegS was compared with the activity of *P. mirabilis* (*Pm*) DegQ and DegS. *Ep*DegP and *Pm*DegQ were detected using polyclonal antibodies. **c** The selective activity of *Ep*DegS was shown in in vitro cleavage experiments using 7 μg *Ep*DegS and 9 μg recombinant RseA (rRseA) as a substrate. To stimulate the activity of *Ep*DegS, 100 μM YFF activator peptide or equal amounts of diluent (−) were added as indicated. 300 ng rEcad was included in the reactions where indicated. Aliquots of samples were analyzed by Western blotting to detect E-cadherin (*upper panel*) and the remaining sample was separated by SDS PAGE following coomassie staining to detect the degradation of RseA (*middle panel*) and *Ep*DegS proteins (*lower panel*). The *asterisk* (*) indicates GST protein co-purified with the *Ep*DegS protein

Since it is unclear whether HtrA homologs have overlapping functions in bacteria, we compared the E-cadherin cleavage activity of the HtrA homologs DegP, DegQ and DegS from EPEC and *P. mirabilis*. Both, DegP and DegQ proteins, but not DegS or the corresponding inactive mutants from EPEC and *P. mirabilis* were caseinolytically active (Additional file 1: Figure S3A and Additional file 1: Figure S3B). Comparing the E-cadherin cleavage activity of the EPEC HtrA proteins DegP, DegQ and DegS revealed that *Ep*DegP cleaved E-cadherin more efficiently than *Ep*DegQ. Compared to *Ep*DegP, *Ep*DegQ induced weak fragmentation of E-cadherin in vitro. *Ep*DegS did not mediate E-cadherin cleavage. *Hp*HtrA was used as a positive control. The polyclonal anti-*Ep*DegP antibody detected *Ep*DegP^wt^ and *Ep*DegP^SA^ and showed weak cross-reactivity to *Ep*DegQ and *Ep*DegS (Fig. 5a). To underline the finding that DegQ proteases also cleave E-cadherin, we compared the E-cadherin cleavage activity of *Ep*DegP, *Ep*DegQ, *Ep*DegS with *Pm*DegQ and *Pm*DegS. In fact, *Pm*DegQ directly cleaved rEcad, which was comparable to *Ep*DegP and *Ep*DegQ (Fig. 5b). Corresponding to *Ep*DegS, *Pm*DegS did not fragment rEcad (Fig. 5b). It has been demonstrated that DegS activity requires stimulation by activator peptides [40]. The fact that recombinant DegS from EPEC is an active protease was demonstrated in an in vitro cleavage experiment using the DegS substrate RseA (Fig. 5c). Upon stimulation with the YFF activator peptide [40], *Ep*DegS efficiently degraded RseA (Fig. 5c, middle panel). In parallel, rEcad was added as indicated. However, rEcad was not targeted by active DegS (Fig. 5c, upper panel). These data underline that E-cadherin shedding is mainly mediated by bacterial DegP and DegQ homologs, while activated DegS failed to target E-cadherin as a substrate.

## Discussion

HtrA proteases are crucially important for bacterial pathogenesis. Their periplasmic chaperone functions facilitate bacterial viability and survival by refolding and degradation of misfolded proteins under stress conditions [1, 3]. Furthermore, HtrA proteins are also implicated in the modulation of pathogen-host interaction by processing of surface-presented virulence factors or adhesins [14, 16, 17]. Another important function was observed for secreted or outer-membrane vesicle-associated HtrA from *H. pylori* and *C. jejuni*, which directly cleaves the extracellular domain of E-cadherin on host cells [15, 27, 29, 30]. HtrA-mediated E-cadherin cleavage opens intercellular adherens junctions allowing bacterial transmigration across the epithelial barrier [15, 29, 30, 33]. Cleavage of E-cadherin has been additionally observed for HtrA expressed by EPEC and *S. flexneri* during infection of cultured epithelial cells and in vitro [15] indicating that HtrA-induced E-cadherin shedding represents a prevalent mechanism in bacterial pathogenesis. In contrast to *H. pylori* or *C. jejuni*, many pathogens express more than one HtrA homolog, namely DegP, DegQ and DegS and it is completely unclear, which of these homologs target E-cadherin. Therefore, we investigated the cleavage activity of the three different bacterial HtrA homologs and found that (i.) additional Gram-negative pathogens *S.* Typhimurium, *Y. enterocolitica* and *P. mirabilis* express E-cadherin-fragmenting HtrA proteases and (ii.) that DegP and DegQ homologs, but not DegS, cleave E-cadherin.

The finding that DegP and DegQ, but not DegS, are active E-cadherin proteases is interesting since it indicates a specific and economical mechanism through which bacteria can interfere directly with host cells functions. Generally, the amino acid sequences of DegP and DegQ proteases show high similarities indicating conserved roles in bacteria. Sequence alignment revealed that *Hp*HtrA lacks 22 amino acids in the LA loop leading to the assumption it could be a DegQ homolog rather than a DegP protein. However, *Hp*HtrA exhibits a higher similarity with DegP proteins. Therefore, it remains vague whether *Hp*HtrA represents a DegP or DegQ protein. From the literature, it is apparently not clear whether HtrA homologs have redundant functions. Consistently described, deletion of *degP* led to a higher sensitivity of the bacteria toward elevated temperatures [43–45]. It has been previously suggested that DegQ can compensate for lacking DegP functions [10]. In other studies, *degP*, *degQ* and *degS* mutants did not show the same phenotype [46] suggesting that the HtrA homologs have different roles. Further, DegQ or DegS re-expression did not fully replace DegP functions in a knock-out mutant [8, 11] implying that DegP and DegQ have different roles in the bacterial periplasm. In our report, we found that only DegP from *H. pylori*, EPEC, *S.* Typhimurium, *Y. enterocolitica*, and DegQ proteases expressed by EPEC and *P. mirabilis* target E-cadherin as a substrate. Since these pathogens interfere with host cell functions via different mechanisms, it needs to be investigated in future, how HtrA-mediated E-cadherin cleavage contributes to the infections with the individual pathogens. Importantly, the opening of the intercellular space can facilitate the contact between pathogens and basolaterally expressed host factors or cells of the immune system. Interestingly, *P. mirabilis* does not express a DegP protein, but an extremely active DegQ protein. Furthermore, active DegP and DegQ proteases also induced a similar fragmentation pattern of E-cadherin indicating that they target identical calcium binding and substrate recognition sites, which have been recently identified for *Hp*HtrA [33, 47]. DegS proteases from EPEC and *P. mirabilis* failed to cleave E-cadherin in vitro. The domain architecture of the DegS proteins differs considerably. A transmembrane domain was predicted in *Pm*DegS, while *Ep*DegS contains a putative signal peptide. Following the highly conserved protease domain, DegS proteins harbor only one PDZ domain [1, 3]. The fact that DegS did not cleave E-cadherin leads to the hypothesis that either the variation on the N-terminus or the second PDZ domain is implicated in the recognition and/or binding of E-cadherin. Based on these observations, we conclude that DegP and DegQ proteins, but not DegS exhibit an E-cadherin-cleaving activity. Our findings were mainly obtained from in vitro experiments as bacterial pathogens harboring genomic deletions of the individual *degP*, *degQ* and *degS* genes are not available to investigate the individual impact of HtrA proteins on bacterial pathogenesis. Still, in infection experiments using Gram-negative pathogens, which express different combinations of DegP, DegQ, and/or DegS, it became apparent that (i.) pathogens do not need DegS and (ii.) pathogens require at least DegP or DegQ for efficient E-cadherin cleavage.

## Conclusions

E-cadherin cleavage during infection has been described for *H. pylori*, *C. jejuni*, EPEC and *S. flexneri* [15, 27, 29]. In this study, we added *S.* Typhimurium, *Y. enterocolitica*, and *P. mirabilis* to the collection of E-cadherin-targeting pathogens. Those gastrointestinal bacteria colonize the epithelium of the intestine as the first barrier. E-cadherin shedding could promote bacterial virulence of these pathogens through providing entry through the polarized epithelium where specific virulence and pathogenic factors then interfere with host cell functions [31, 32]. Hence, it would be highly interesting to investigate the influence of the different HtrA homologs in their respective experimental animal models in vivo as HtrA proteins represent attractive therapeutic target molecules. The finding that the uropathogen *P. mirabilis* also induces E-cadherin shedding through its highly active DegQ protein also suggests a possible role for HtrA proteins in pathogens, which colonize non-intestinal epithelia. Therefore, future studies are necessary to study the function of HtrAs during the colonization of pathogens targeting the epithelium of other organs.

## Additional file

Additional file 1: Show an additional alignment of DegQ and DegS proteins (**Figure S1**), validation of anti-HtrA antibodies (**Figure S2**), and the activity of recombinant proteases (**Figure S3**). (PDF 1620 kb)

## Abbreviations

DegP/Q/S: Periplasmic serine endoproteases
Dlg1: *Drosophila* disc large tumor suppressor
EPEC: Enteropathogenic *escherichia coli*
HtrA: High temperature requirement A
NTF: N-terminal fragment
PDZ: Postsynaptic density protein (PSD95)
ZO-1: Zonula occludens-1 protein
