# Supplementary material for: HtrA-mediated E-cadherin cleavage is limited to DegP and DegQ homologs expressed by gram-negative pathogens
Source: Cell Commun Signal. 2016 Dec 8;14:30. doi: 10.1186/s12964-016-0153-y (PMC5146865; doi:10.1186/s12964-016-0153-y)
Supplement: Additional file 1: — Show an additional alignment of DegQ and DegS proteins (Figure S1), validation of anti-HtrA antibodies (Figure S2), and the activity of recombinant proteases (Figure S3). (PDF 1620 kb) [file 12964_2016_153_MOESM1_ESM.pdf]

HtrA-mediated E-cadherin cleavage is limited to DegP and DegQ homologs expressed by Gram-negative pathogens

Carmen M. Abfalter[#], Maria Schubert[#], Camilla Götz, Thomas P. Schmidt, Gernot Posselt, Silja Wessler*

Division of Microbiology, Department of Molecular Biology, Paris-Lodron University of Salzburg, Billroth Str. 11, A-5020 Salzburg, Austria.

*Corresponding author: Silja Wessler, Division of Molecular Biology, Paris-Lodron University, Salzburg, Austria. E-mail: silja.wessler@sbg.ac.at, Tel.: +43 662 8044 7210, Fax: +43 662 8044 7209.

Figure legends

Figure S1. Sequence alignment of the DegP homologs DegQ and DegS from EPEC and *P. mirabilis*. (A) Signal peptide (orange), proteolytic domain (green) with the catalytic triad (red) and two PDZ domains (purple) of *Ep*DegQ and *Pm*DegQ. **(B)** Signal peptide (orange), transmembrane domain (red) proteolytic domain (green) with the catalytic triad (red) and one PDZ domain (purple) of *Ep*DegS and *Pm*DegS.

Figure S2. Polyclonal antibodies recognizing different HtrA homologs. The specificity of antibodies generated for the detection of recombinant *Ep*DegP, *St*HtrA, *Ye*DegP and *Pm*DegQ was demonstrated by Western blotting.

Figure S3. Recombinant DegP and DegQ, but not DegS are caseinolytically active. (A) Equal protein amounts of recombinant *Ep*DegP and *Ep*DegQ wildtype (wt) and their corresponding inactive mutants (SA) indicated caseinolytic activity (upper panel) in zymography analyses. Activity of *Ep*DegS (wt) and the inactive mutant (SA) was not detectable. In parallel, proteins were separated by SDS PAGE followed by coomassie-staining (lower panel). **(B)** Comparison of recombinant *Ep*DegP, *Ep*DegQ and *Ep*DegS with *Pm*DegQ and *Pm*DegS indicated different levels of activity in casein zymography (upper panel). Proteins were separated by SDS PAGE and coomassie-stained to demonstrate equal protein loading (lower panel).

Figure S1

A

```
EpDegQ         --MKKQTQLLSALALSVGLTLSAS-FQAVASIPGQVAAQAPLPSLAPMLEKVLPAVVSVR
PmDegQ         MLTKKRKLLLSALAMSVGLSLSTLPATSMAALPSVMPSGEQLPSLAPMLEKVLPAVVSVH
                   **:. ******:****:**:     ::*::*. : :    ******************:

EpDegQ         VEGTASQGQKIPEEFKKFFGDDLPDQ--PAQPFEGLGSGVIINANKGYVLTNNHVINQAQ
PmDegQ         VSGTRVQSQQVPEEFKFFFGPNFPSQQQSIRPFEGLGSGVIIDAEKGYVLTNNHVVDGAD
               *.**  *.*::***** *** ::*.*     :***********:*:**********:: *:

EpDegQ         KISIQLNDGREFDAKLIGSDDQSDIALLQIQNPSKLTQIAIADSDKLRVGDFAVAVGNPF
PmDegQ         KIQLQINDGREFDAKLIGSDPQTDIALLQIEKPNNLTAIKMTDSDKLRVGDFAVAVGNPF
               **.:*:************** *:*******::*.:** * ::******************

EpDegQ         GLGQTATSGIISALGRSGLNLEGLENFIQTDASINRGNSGGALLNLNGELIGINTAILAP
PmDegQ         GLGQTATSGIISALGRSGLNLEGLENFIQTDASINRGNSGGALVNLNGELIGINTAILAP
               ******************************************:****************

EpDegQ         GGGSVGIGFAIPSNMARTLAQQLIDFGEIKRGLLGIKGTEMSADIAKAFNLDVQRGAFVS
PmDegQ         GGGNIGIGFAIPSNMARDLSQQLITHGEVKRGILGIRGTEMNADLAKSFKIDAQRGAFVS
               ***.:************ *:****  .**:***:****:****.**:**:*::*.*******

EpDegQ         EVLPGSGSAKAGVKAGDIITSLNGKPLNSFAELRSRIATTEPGTKVKLGLLRNGKPLEVE
PmDegQ         EVLADSSAAKAGIKPGDVLISIDGKRINSFAELRAKVGTTPPGKEILIGLIRQGKPMDVK
               ***  *.:****:* **:: *::** :*******:::.** **.:: :**:*:***::*:

EpDegQ         VTLDTSTSSSASAEMITPALEGATLSDGQLKDGGKGIKIDEVVKGSPAAQAGLQKDDVII
PmDegQ         VTLEKQSADATRADNFSPALQGATLSNYV-NKQVKAVAVDSVEKGSAAAASGLQKGDLII
               ***:..:.:.: *: ::***:*****:   :.  *.: :*.* *** **  :**** *:**

EpDegQ         GVNRDRVNSIAEMRKVLAAKPAIIALQIIRGNESLYLLMR----
PmDegQ         GINNMPIGSLGDLRKTIDAKPPVLALNIKRGNEEIYLLLRNSSR
               *:*.  : *:.::**.: ***  ::**:* ****.:***:*
```

B

```
EpDegS         MFVKLLRSVAIGLIVGAILLVAMPSLRSLNPLSTPQFDSTDETPASYNLAVRRAAPAVVN
PmDegS         MLSKLLRSTLIGVLTAAILLVAVPSIRPIFIEHLI-NGDFLNAPFSYNKAVRRAAPAVVN
               *: *****. **::..******:**:* :          .  ::* *** ***********

EpDegS         VYNRGLNTNSHNQLEIRTLGSGVIMDQRGYIITNKHVINDADQIIVALQDGRVFEALLVG
PmDegS         VYSSTMGSFSQEGRELTSLGAGVIMDPRGYILTNQHVINNADQIIVALQNGDLYEGLLIG
               **.  : : *::  *: :**:***** ****:**:****:*********:* ::*.**:*

EpDegS         SDSLTDLAVLKINATGGLPTIPINPRRVPHIGDVVLAIGNPYNLGQTITQGIISATGRIG
PmDegS         SDPLTDLAVLKVDA-EKLPTIPINSKRISHVGDVVLAIGNPFNIGQTITQGIISATGRVG
               ** ********::*   ******* :*: *:**********:*:**************:*

EpDegS         LNPTGRQNFLQTDASINHGNSGGALVNSLGELMGINTLSFDKSND---GETPEGIGFAIP
PmDegS         LSPTRYQNFLQTDASINEGNSGGALINTEGELVGINTMTFDSGTYNGYRPSAEGLGFAIP
               *.**  ***********.*******:*: ***:****::**...        : **:*****

EpDegS         FQLATKIMDKLIRDGRVIRGYIGIGGREIAPLHAQSGGMDQLQGIVVNEVSPDGPAANAG
PmDegS         TQLAVKIMNKLIRDGRVIRGFIGITAKELPKIRSSNTDIKQIQGLRIFRITPNSPADKAG
                ***.***:**********:*** .:*:  ::::.  :.*:**: : .::*:.** :**

EpDegS         IQVNDLIISVNNKPAISALETMDQVAEIRPGSVIPVVVMRDDKQLTLQVTIQEYPATN
PmDegS         MKIGDIILSIDHAPAKSAMEMMDYIAETRPGTVLPVTLLRDGNEINVDVTISEYQPES
               ::: *:*:*::. ** **:* ** :** ***:*:**.::**.::::.::***.**   .
```

Figure S2

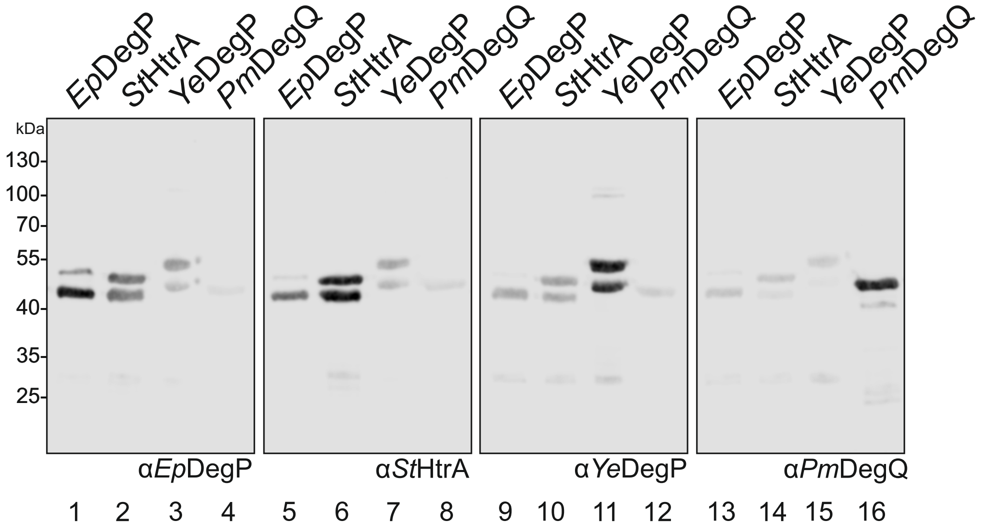

EpDegP
StHtrA
YeDegP
PmDegQ
EpDegP
StHtrA
YeDegP
PmDegQ
EpDegP
StHtrA
YeDegP
PmDegQ
EpDegP
StHtrA
YeDegP
PmDegQ
kDa
130
100
70
55
40
35
25
αEpDegP
αStHtrA
αYeDegP
αPmDegQ
1 2 3 4 5 6 7 8 9 10 11 12 13 14 15 16

Figure S3

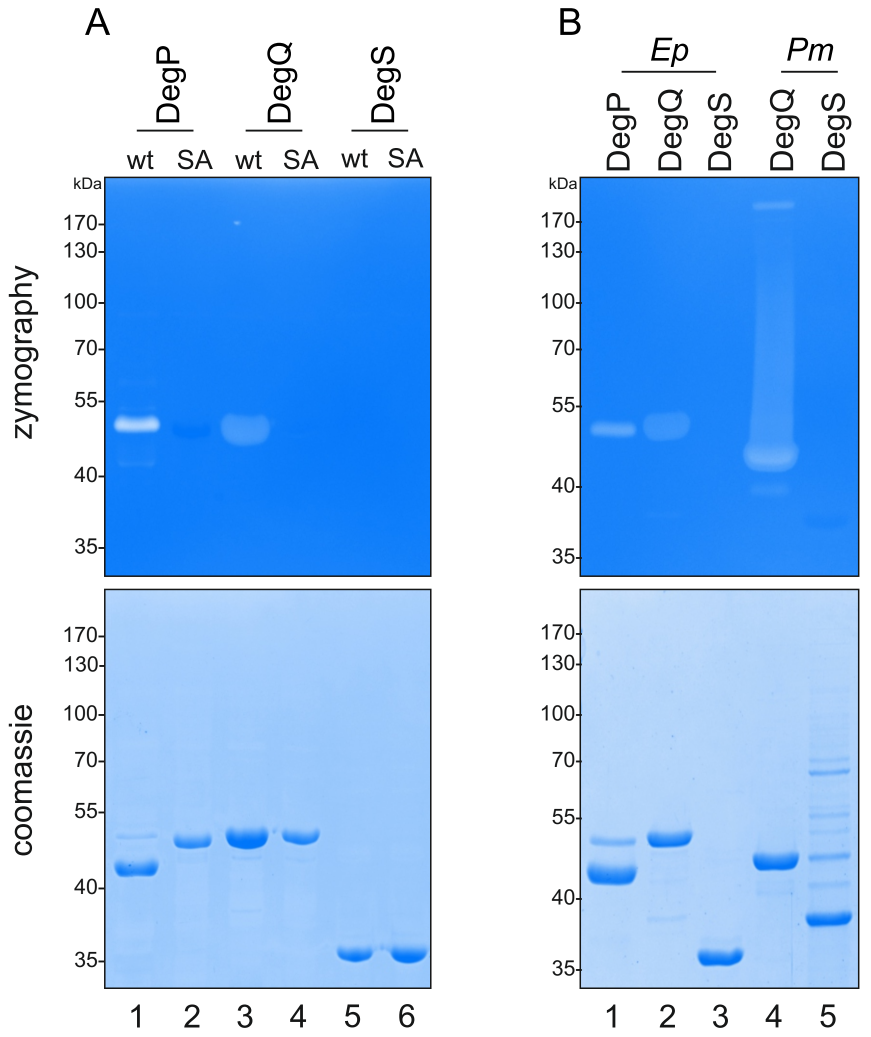

A
DegP
DegQ
DegS
wt SA wt SA wt SA
kDa
170
130
100
70
55
40
35
zymography
coomassie
1 2 3 4 5 6
B
Ep
Pm
DegP DegQ DegS DegQ DegS
1 2 3 4 5
